# Circulating tumor DNA as a prognostic indicator in resectable pancreatic ductal adenocarcinoma: A systematic review and meta-analysis

**DOI:** 10.1038/s41598-019-53271-6

**Published:** 2019-11-18

**Authors:** Jee-Soo Lee, Tae-Min Rhee, Daniel Pietrasz, Jean-Baptiste Bachet, Pierre Laurent-Puig, Sun-Young Kong, Erina Takai, Shinichi Yachida, Tatsuhiro Shibata, Jung Woo Lee, Hyoung-chul Park, Dae Young Zang, Kibum Jeon, Jiwon Lee, Miyoung Kim, Han-Sung Kim, Hee Jung Kang, Young Kyung Lee

**Affiliations:** 1 Department of Laboratory Medicine, Hallym University Sacred Heart Hospital, Anyang, Korea; 2 Department of Internal Medicine, Seoul National University Hospital, Seoul, Korea; 3 Université Paris Sorbonne Cité, Centre Universitaire des Saints-Péres, Paris, France; 4 Sorbonne Université, Hôpitaux Universitaires Pitié-Salpétrière, APHP, Paris, France; 5 University Paris Descertes, UMR-S1147 Paris, France; 6 Department of Laboratory Medicine, Center for Diagnostic Oncology, National Cancer Center, Goyang, Korea; 7 Division of Cancer Genomics, National Cancer Center Research Institute, Tokyo, Japan; 8 Department of Surgery, Hallym University Sacred Heart Hospital, Anyang, Korea; 9 Center for Colorectal Cancer, Research Institute and Hospital, National Cancer Center, Goyang, Korea; 10 Department of Internal Medicine, Hallym University Sacred Heart Hospital, Anyang, Korea

**Keywords:** Pancreatic cancer, Prognostic markers, Cancer genomics, Cancer genetics, Pancreatic cancer

## Abstract

Circulating tumor DNA (ctDNA) is a promising prognostic biomarker in various cancers. Due to the high recurrence rate of resectable pancreatic ductal adenocarcinoma (PDAC), effective strategies for prognostic stratification are necessary. Yet, for resectable PDAC, prognostic impact of ctDNA lacks systemic evidence. We sought to investigate the prognostic significance of baseline ctDNA and postoperative ctDNA in patients with resectable PDAC. PubMed, EMBASE, and the Cochrane library were searched up to March 2019. Five studies met the inclusion criteria, and 375 patients were pooled for the meta-analysis. Positive ctDNA significantly indicated poor overall survival (at baseline, hazard ratio [HR] 2.27, 95% confidence interval [CI] 1.13–4.56; postoperative, HR 3.66, 95% CI 1.45–9.28). Patients with detectable ctDNA showed the trend to have higher risk for disease recurrence than those without detectable ctDNA (at baseline, HR 1.96, 95% CI 0.65–5.87; postoperative, HR 2.20, 95% CI 0.99–4.87). The results were consistent regardless of pre- or post-operative ctDNA. There was no significant heterogeneity among the included studies. In conclusion, our meta-analysis revealed that ctDNA, either at baseline or postoperative, might be a useful prognostic biomarker for stratifying risk of death and recurrence in resectable PDAC.

## Introduction

Pancreatic ductal adenocarcinoma (PDAC) is one of the most lethal cancers, where the five-year survival rate for all stages of PDAC as low as 6–8%^1^. Surgical resection remains the only chance for cure, increasing the five-year survival rate to 15–25%^2^. However, tumors recur in 85% of resected cases; therefore, identifying patients with a high risk of recurrence is a major challenge^3^. Hence, it is important to find out effective strategies for evaluating the risk of recurrence and mortality in resectable PDAC.

Circulating tumor DNA (ctDNA) is a promising blood-based biomarker in cancer management^4,5^. The majority of previous reports have summarized the benefit of ctDNA as a non-invasive marker for treatment selection, real-time disease monitoring, detection of residual disease, and estimation of prognosis^6–8^. Recently, there has been increasing attention towards the emerging role of ctDNA in early-stage cancers; pre- and post-operative ctDNA in various cancers has been introduced as a useful prognostic biomarker to indicate recurrence after resection^9^. Regarding PDAC, a few studies have focused on the prognostic value of ctDNA in patients undergoing curative resection^7,10^. Yet, the results have been controversial, and there is lack of evidence that systematically demonstrates the prognostic value of ctDNA in resectable PDAC.

Therefore, we performed a meta-analysis to evaluate the prognostic significance of ctDNA in patients with resectable PDAC in terms of overall survival (OS) and disease-free survival (DFS).

## Results

### Search results

**Figure 1:**
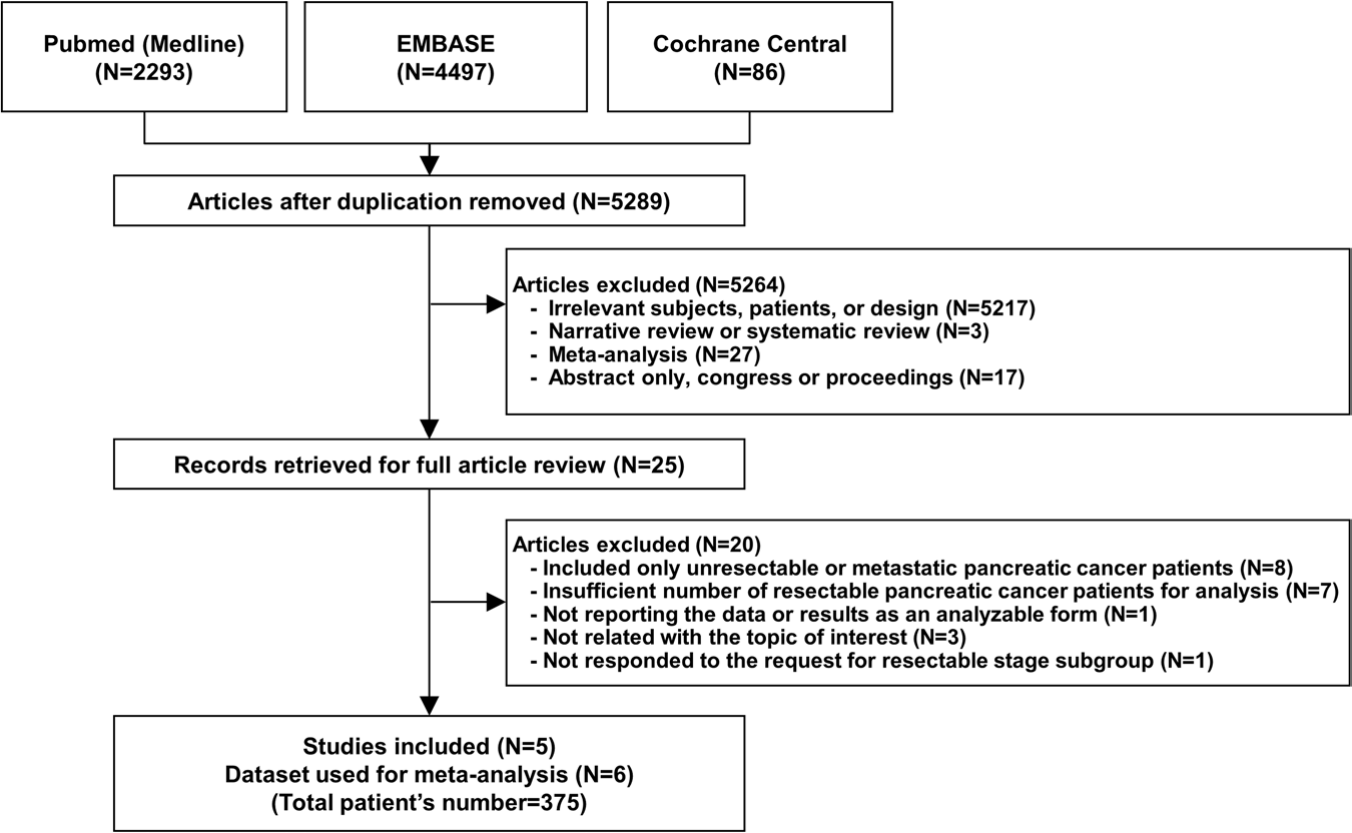
Flow diagram of study selection. The flow diagram is presented according to the Preferred Reporting Items for Systematic Reviews and Meta-Analyses (PRISMA) guidelines.

A total of 5,289 citations were identified after removing duplicates. Among these, 25 articles were retrieved for a full review, and five were selected for the analysis (Fig. 1). The characteristics of the 20 studies excluded after the full review are summarized in the Supplementary Material. The final five studies with six results encompassed 375 patients with resectable PDAC grouped based on the time point of measuring ctDNA (patients measured with baseline sample, n = 299; patients measured with postoperative sample, n = 76). Among the six results, four results provided HRs and 95% CIs in patients with detectable ctDNA at baseline, and the other two results provided HRs and 95% CIs in patients with detectable ctDNA after surgery.

### Study characteristics and risk of bias within studies

The main characteristics of the individual studies are summarized in Table 1 and in Supplementary Table 1. All studies included a significant proportion of resectable patients, and per our request, all authors provided survival data of patients in the resectable stage. The positive ctDNA detection rate varied among studies from 8.33% to 68.57%. Three studies confirmed *KRAS* mutation status with matched tissue where the prevalence of *KRAS* mutations ranged from 81.9% to 94.3%. Except one study that performed Peptide Nucleic Acid-directed PCR clamping analysis using serum samples, all studies performed either dPCR or NGS using plasma samples. Mean or median age ranged from 66 to 70 years, and the proportion of males from 52.4 to 64.4%. Four studies analyzed Asian populations. All studies were retrospective investigations, while four provided adjusted HRs and 95% CIs using multivariable regression.

**Table 1:** Characteristics of studies selected for analysis.

Source (Year)	Study period	Patient No.	Time point measuring ctDNA	ctDNA (+) rate	Follow-up duration	Median survival	Sample type	Detection method	Platform	Target mutation	Age (Y)	Male (%)	Ethnicity	Tissue mutation
Takai *et al*.^23^	2011–2014	108	Baseline	8.33%	Up to 40mo	NR	Plasma	Digital PCR	RainDrop	*KRAS* - G12D - G12R - G12V - G13D	66	61.8	Asian	NA
Hadano *et al*.^7^	2007–2013	105	Baseline	31.43%	14–96mo (mean 54mo)	13.6mo/27.6mo	Plasma	ddPCR	Bio-Rad QX100	*KRAS* - G12D - G12V - G12R	69	52.4	Asian	81.9% (86/105)
Pietrasz *et al*.^8^	2011–2015	31	Postoperative	19.35%	Median 33.3mo	19.3mo/32.2mo	Plasma	Amplicon-based NGS*	Ion Proton	22 genes**	68	54.8	Non-Asian	NA
Kim *et al*.^24^	2015–2017	41	Baseline	68.57%	Median 10.03mo	NR	Plasma	ddPCR	Bio-Rad QX200	*KRAS* - G12A - G12C - G12D - G12R - G12S - G12V - G13D	66	63.2	Asian	94.3% (33/35)^**^
Nakano *et al*.^10^	2013–2016	45	Both baseline and postoperative	24.44% (baseline)/44.44% (post)	Up to 42mo	NR	Serum	PNA-directed PCR clamping		*KRAS* codon 12/13	70	64.4	Asian	83.3% (35/42)

Abbreviations: ctDNA, circulating-tumor DNA; mo, month; NR, not reached; ddPCR, droplet digital PCR; NGS, next-generation sequencing; PNA, peptide nucleic acid.

^*^Sequencing library was prepared using Ion AmpliSeq Colon and Lung Cancer Research Panel v2 (Thermo Fisher). The libraries were processed on Ion Chef system and sequenced on the Ion Proton system.

^**^KRAS, *EGFR*, *BRAF*, *PIK3CA*, *AKT1*, *ERBB2*, *PTEN*, *NRAS*, *STK11*, *MAP2K1*, *ALK*, *DDR2*, *CTNNB1*, *MET*, *TP53*, *SMAD4*, *FBX7*, *FGFR3*, *NOTCH1*, *ERBB4*, *FGFR1* and *FGFR2*.

The quality of studies included was assessed via NOS for non-randomized studies and the results are presented in Supplementary Table 2. All five studies received at least seven stars, and thus fulfilled the adequacy criteria for non-randomized studies.

### Impact of ctDNA detection on the prognosis of resectable PDAC

**Figure 2:**
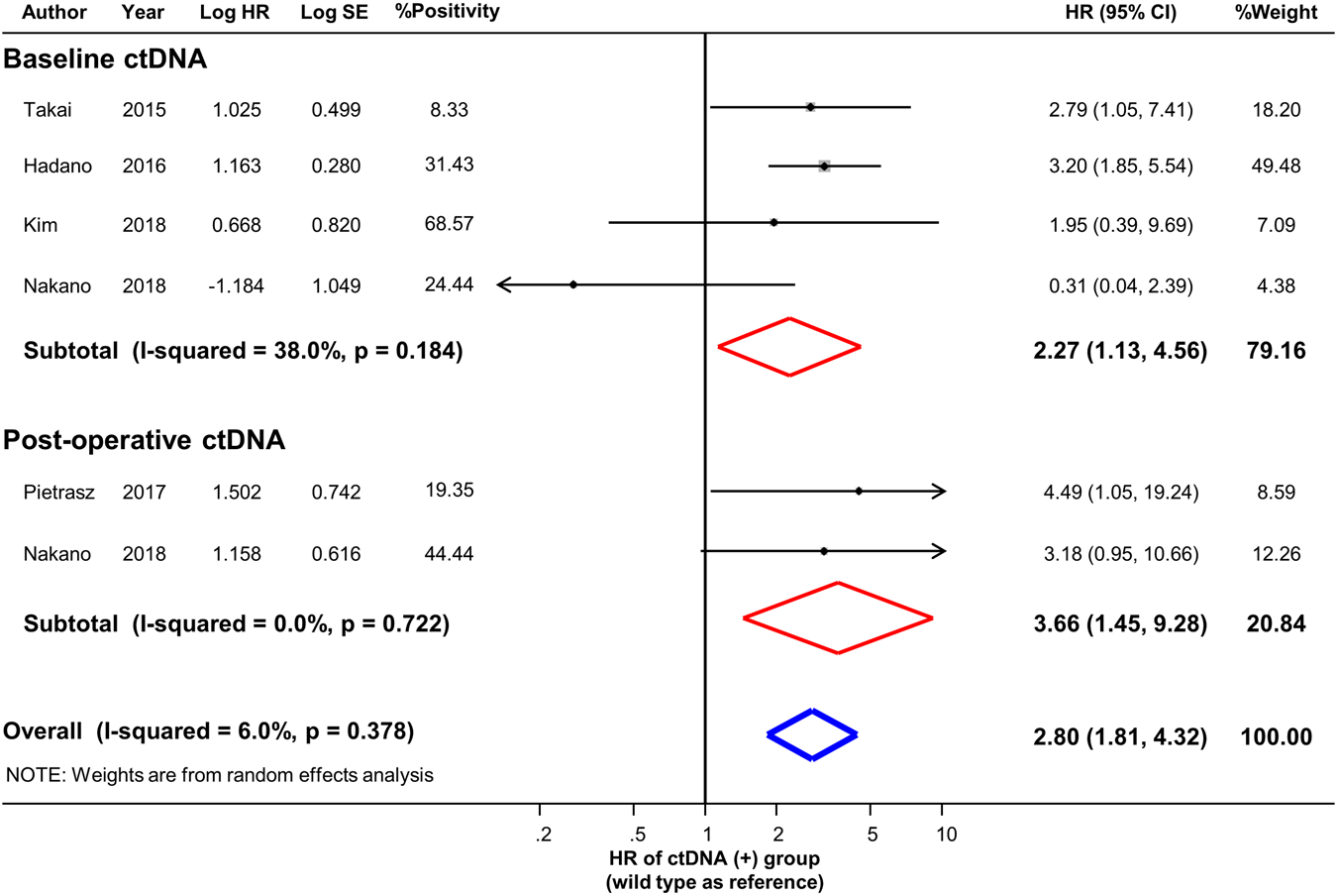
Prognostic effect of baseline or post-operative ctDNA in resectable PDAC. Hazard ratios with 95% confidence intervals are displayed by individual studies, describing pooled overall effects for baseline ctDNA and for post-operative ctDNA, respectively. Abbreviations: HR, hazard ratio; SE, standard error; CI, confidence interval.

**Figure 3:**
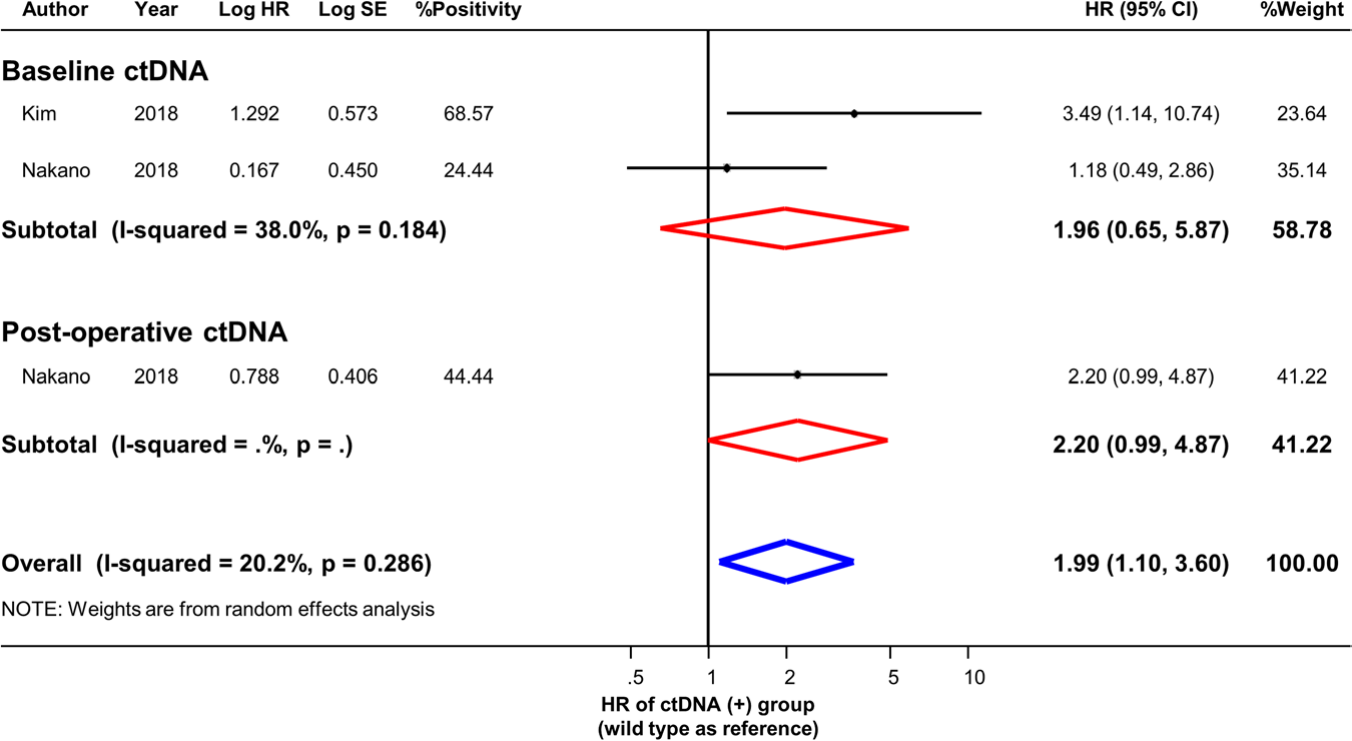
Effect of ctDNA on disease-free survival in resectable PDAC. Hazard ratios with 95% confidence intervals are displayed by individual studies, describing pooled overall effects for baseline ctDNA and for post-operative ctDNA, respectively. Abbreviations: HR, hazard ratio; SE, standard error; CI, confidence interval.

Six study results (four using preoperative sample, and two using post-operative sample) indicated the association of ctDNA detection with OS, three (two using preoperative sample, and one using post-operative sample) of which evaluated the association of ctDNA with DFS. Pooled results from the random-effects models of OS and DFS are presented in Figs 2 and 3, respectively. The risk was significantly higher in the ctDNA-positive group than in the ctDNA-negative group in terms of mortality, regardless of the time point (at baseline or postoperative) of sample collection (detectable baseline ctDNA, pooled HR 2.27, 95% CI 1.13–4.56; detectable postoperative ctDNA, pooled HR 3.66, 95% CI 1.45–9.28). The risk for disease recurrence showed the trend of increase in the ctDNA-positive group than in the ctDNA-negative group for both preoperative and postoperative measurement (detectable baseline ctDNA, pooled HR 1.96, 95% CI 0.65–5.87; detectable postoperative ctDNA, pooled HR 2.20, 95% CI 0.99–4.87). The I-squared statistical heterogeneity was not significant for either outcome (OS, baseline ctDNA I^2^ = 38.0%, *p* = 0.184, postoperative ctDNA I^2^ = 0.0%, *p* = 0.722; DFS, baseline ctDNA I^2^ = 38.0%, *p* = 0.184). A funnel plot and results of Egger’s and Begg’s tests demonstrated that there was no significant publication bias in qualitative or quantitative terms (Supplementary Fig. 1).

### Subgroup analysis and meta-regression analysis

**Figure 4:**
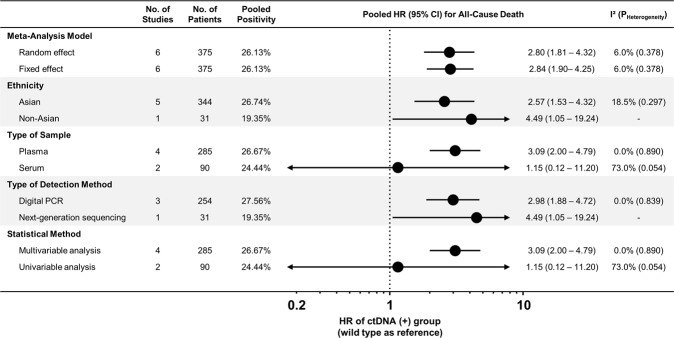
Subgroup analysis. Effect of ctDNA on overall survival according to the various subgroups is presented. Abbreviations: HR, hazard ratio; CI, confidence interval.

The results of subgroup analysis stratified based on several variables are shown in Fig. 4. The pooled results were consistent in both random- and fixed-effects model. Stratification based on ethnicity (Asian or non-Asian population) showed that the prognostic impact of ctDNA on OS was significant both in Asian and non-Asian patients. The source of heterogeneity was identified in the subgroups of specimen type and statistical method. Excluding one study with two results that used serum samples and univariable regression analysis, the pooled results were significant with heterogeneity of I^2^ = 0.0%. In meta-regression analysis, no significant effects were observed based on demographic characteristics including the number of patients, ctDNA positivity rate, age, or proportion of males (Table 2).

**Table 2:** Results of Meta-Regression Analysis.

	Meta-regression OR	95% CI	*P* value
By patient number	1.006	0.974–1.039	0.594
By positivity rate	0.996	0.935–1.062	0.866
By mean/median age	0.822	0.406–1.664	0.442
By proportion of men	0.961	0.812–1.137	0.505

Abbreviations: OR, odds ratio; CI, confidence interval.

## Discussion

In the present meta-analysis, the prognostic significance of ctDNA in baseline and postoperative samples from the patients with resectable PDAC was evaluated, respectively. The results can be summarized as following. (1) Detectable ctDNA in resectable PDAC had a significant negative effect on prognosis in terms of both OS and DFS; (2) These results were consistently observed using either pre- or post-operative ctDNA; (3) No significant heterogeneity was observed among the included studies, and heterogeneity decreased to 0.0% after excluding one study that used serum samples and univariable analysis; (4) There were no differences between the results according to ethnicity, age, sex, rate of positive ctDNA detection, or sample size in subgroup analysis and meta-regression. To the best of our knowledge, this is the first meta-analysis to identify the prognostic role of ctDNA in patients with resectable PDAC.

PDAC is well-known as a digestive system cancer with poor prognosis, and even resectable stage PDAC shows a five-year survival rate of only 15–25%^2^. Patients with early stage PDAC are usually recommended to undergo extensive surgery as well as adjunctive chemotherapy before and/or after surgery. Nevertheless, up to 85% of resectable PDAC results in recurrence^3^ and therefore, the use of biomarkers to predict the prognosis and recurrence of PDAC is essential. Recently, ctDNA has been suggested as a promising prognostic biomarker in various cancers including PDAC. Significant associations between ctDNA and OS/DFS were consistently shown in various studies and meta-analyses^11–13^. In a recent review, Lee *et al*. have documented clinical application of ctDNA in PDAC in various aspects including detection of ctDNA in patients with premalignant lesion (i.e., intraductal papillary mucinous neoplasm), and use of ctDNA as prognostic and predictive markers without distinguishing between resectable and advanced stages^14^. However, most studies to date have focused on patients with advanced or metastatic stage PDAC^15–20^, or heterogeneous populations including a small proportion of early stage PDAC^21–25^. There are only a few studies demonstrating the clinical significance of ctDNA in resectable PDAC, and it is necessary to establish comprehensive evidence in this specific population. Therefore, in this study, we exclusively analyzed patients undergoing curative resection, which provided novel insights into the prognostic significance of ctDNA in early stage PDAC. To incorporate relevant study results to the extent possible, we also collected subgroup data limited to resectable PDAC not reported in the original articles by individually contacting the authors of each study. Thus, incorporating unique data that have not been reported elsewhere, the present study succeeded to show, for the first time, the pooled prognostic effect of detectable ctDNA in resectable PDAC.

The present study highlights the fact that either baseline or postoperative ctDNA can be used as a prognostic indicator in resectable PDAC in terms of OS. We found similar trend for the disease recurrence, although not reaching statistical significance due to the limited number of studies that reported DFS in patients with resectable PDAC. Further studies focusing on the effect of ctDNA on the disease recurrence of early stage PDAC are required to solidify our findings. There is increasing attention towards the emerging role of ctDNA in risk stratification and residual tumor monitoring after curative resection in early stage PDAC patients^26^. It is important to note that if we could identify patients who are at high risk of recurrence at baseline, we can then focus on active surveillance allowing early treatment to prevent or delay recurrence. Furthermore, monitoring ctDNA serially, at multiple time-points after surgery, could be valuable in prediction of clinical outcome and adjusting further treatment strategies. In summary, our results demonstrate that both baseline and post-operative ctDNA can be an important prognostic biomarker if used in parallel or in a complementary. *Nakano et al*. reported contrasting results regarding the association of ctDNA in preoperative serum with OS^10^. We speculate that the different choice of matrix might have contributed to this outcome, because the above study is the only one to use serum. Therefore, we performed a subgroup analysis stratifying the studies based on the specimen type. A considerable difference was found between plasma ctDNA and serum ctDNA; plasma ctDNA was predictive of poor OS without any heterogeneity, but not serum ctDNA. This finding suggests that plasma is an optimal ctDNA source. Notably, during clotting process, leukocyte lysis more frequently occurs in serum than in plasma, possibly leading to dilution of ctDNA with genomic DNA from leukocytes^26,27^. Thus, the mutant allelic fraction of cfDNA in serum may have been underestimated in terms of sensitivity.

Several technologies (e.g., real-time or digital PCR, NGS, and BEAMing [beads, emulsion, amplification, and magnetics]) have been developed for the detection of ctDNA, and each of these technologies has different assay performance characteristics^26^. We attempted to determine if the different assay platforms lead to different prognostic values of ctDNA in resectable PDAC. Our subgroup analysis included three studies that used dPCR (droplet digital PCR, BioRad, n = 2; microfluidic digital PCR, RainDrop^®^, n = 1) and comparison of the pooled result using NGS (Ion Proton^TM^). Although dPCR methods more frequently detected ctDNA (pooled positivity, 27.6%) than the NGS method (pooled positivity, 19.4%), significant prognostic value was achieved with both methods. While dPCR focuses on the detection of rare mutations at specific loci and attains high sensitivity ranging from 0.001% to 0.1%, NGS-based approaches have the potential to detect a broad range of molecular targets (e.g., *TP53*) in PDAC other than *KRAS*^28^. Moreover, advanced NGS strategies that minimize artifacts by using molecular barcodes or digital error suppression enable highly sensitive detection of ctDNA with allele frequencies theoretically ranging from 0.00025% to 0.1%^29,30^. We speculate that these technologies may provide benefits in early stages of cancer (e.g., MRD monitoring and early diagnosis of relapse)^31^. Further studies may be needed to verify the prognostic significance of ctDNA in resectable PDAC, using advanced NGS strategies.

The present meta-analysis has several limitations. First, the selected studies varied in their clinical and methodological characteristics. Second, the number of included studies and pooled patients were relatively small. However, despite using a limited number of studies on ctDNA in resectable PDAC, our results were consistent without any significant heterogeneity. Third, although four out of five studies provided multivariable-adjusted results, the effect of unmeasured confounders could exist due to non-randomized baseline characteristics.

In conclusion, our meta-analysis demonstrated the significant prognostic impact of detectable ctDNA (either at baseline or postoperative) in resectable PDAC. Our findings suggest that ctDNA might be a useful predictive biomarker of both mortality and recurrence in resectable PDAC.

## Methods

### Data sources and searches

We conducted systematic searches of the databases PubMed, EMBASE, and the Cochrane Central Register of Controlled Trials for published or unpublished studies. A manual search of references cited in articles, recent reviews, editorials, and meta-analyses was performed. We did not apply any restrictions on the language, study period, or sample size during the search process.

### Study selection and outcome definition

The eligibility criteria for study selection were 1) execution until March 2019, 2) inclusion of patients with resectable PDAC, 3) ctDNA assessment using plasma or serum, and 4) hazard ratios (HR) and 95% confidence intervals (CI) of survival outcomes in resectable groups being directly accessible, statistically estimable, or available from the authors on request. We excluded studies that included only patients with unresectable or metastatic cancer, had a small number of patients (n < 30) insufficient for statistical analysis, or did not report data or results in an analyzable form. Two investigators independently screened titles and abstracts, identified duplicates, reviewed full articles, and determined their eligibility. Disagreements were resolved via discussion. The last search was performed in March 2019. The primary outcome was OS defined as the interval from the defined initiation in each study to death, and the secondary outcome was DFS calculated from the initiation to recurrence.

### Data extraction and quality assessment

We extracted the following characteristics from each eligible study; study design, number of patients who underwent curative resection, ctDNA positivity rate, duration of follow-up, specimen type, method/platform of ctDNA detection, target genes/variants, patient demographics, and HR with 95% CI in the ctDNA-positive group (with the ctDNA-negative group as reference). Of note, one study assessed ctDNA at two different time points (at baseline and after surgery) and we incorporated both results in the analysis (five studies with six results). For the studies that reported survival outcomes irrespective of clinical stage, we contacted each corresponding author individually and requested the adjusted HRs and 95% CIs in the subgroup limited to the resectable stage. The quality of eligible studies was assessed using the Newcastle–Ottawa Scale (NOS) checklist for non-randomized studies.

### Data synthesis and analysis

Random-effect models were applied for all analyses, and pooled HRs with 95% CIs were represented as statistical summaries. The pooled HRs and 95% CIs of the random- and fixed-effects models were calculated using the restricted maximum likelihood (REML) and Mantel–Haenszel methods.

We quantified the statistical heterogeneity using I^2^ statistics. Publication bias was assessed through funnel plot asymmetry using Egger’s and Begg’s tests. Meta-regression analysis was performed to assess the relationship between effect size (log-ORs) and patient number, ctDNA positivity rate, mean or median age, and proportion of males.

To gain insights into prognostic significance of sampling time points, we assessed the HRs of detectable baseline ctDNA and of detectable postoperative ctDNA, respectively. Subgroup analyses were performed as follows to explore the source of heterogeneity; (1) meta-analysis model (random vs. fixed effects); (2) ethnicity of the study population (Asian vs. Non-Asian); (3) specimen type used in the study (plasma vs. serum); (4) detection method (digital PCR [dPCR] vs. next-generation sequencing [NGS]); and (5) statistical method used in the study (multivariable vs. univariable regression analysis). Two-sided *p*-values < 0.05 were considered to be statistically significant. Statistical computations used the standard software STATA/SE v12.0 (StataCorp LP, College Station, Texas, USA). The present study followed the Preferred Reporting Items for Systematic Reviews and Meta-Analyses (PRISMA) guidelines (Supplementary Table 3) and the Meta-analysis of Observational Studies in Epidemiology (MOOSE) guidelines.

## Supplementary information

Supplementary Material
