# Supplementary material for: Circulating tumor DNA as a prognostic indicator in resectable pancreatic ductal adenocarcinoma: A systematic review and meta-analysis
Source: Sci Rep. 2019 Nov 18;9:16971. doi: 10.1038/s41598-019-53271-6 (PMC6861312; doi:10.1038/s41598-019-53271-6)
Supplement: Supplementary file 1 — Supplementary Material [file 41598_2019_53271_MOESM1_ESM.pdf]

Circulating tumor DNA as a prognostic indicator in resectable pancreatic ductal adenocarcinoma: A systematic review and meta-analysis

Jee-Soo Lee, MD,[1] Tae-Min Rhee, MD,[2] Daniel Pietrasz, MD,[3] Jean-Baptiste Bachet, MD, PhD,[4] Pierre Laurent-Puig, MD, PhD,[5] Sun-Young Kong, MD, PhD,[6] Erina Takai, PhD,[7] Shinichi Yachida, MD, PhD,[7] Tatsuhiro Shibata, MD, PhD,[7] Jung Woo. Lee, MD,[8] Hyoung-chul Park, MD, PhD,[9] Dae Young Zang, MD, PhD,[10] Kibum Jeon, MD,[1] Jiwon Lee, MD,[1] Miyoung Kim, MD, PhD,[1] Han-Sung. Kim, MD, PhD,[1] Hee Jung Kang, MD, PhD,[1] Young Kyung Lee, MD, PhD,[1]

[1]Department of Laboratory Medicine, Hallym University Sacred Heart Hospital, Anyang, Korea; [2]Department of Internal Medicine, Seoul National University Hospital, Seoul, Korea; [3]Université Paris Sorbonne Cité, Centre Universitaire des Saints-Péres, Paris, France; [4]Sorbonne Université, Hôpitaux Universitaires Pitié-Salpétrière, APHP, Paris, France; [5]University Paris Descertes, UMR-S1147, Paris, France; [6]Department of Laboratory Medicine, Center for Diagnostic Oncology, National Cancer Center, Goyang, Korea; [7]Division of Cancer Genomics, National Cancer Center Research Institute, Tokyo, Japan; [8]Department of Surgery, Hallym University Sacred Heart Hospital, Anyang, Korea; [9]Center for Colorectal Cancer, Research Institute and Hospital, National Cancer Center, Goyang, Korea; [10]Department of Internal Medicine, Hallym University Sacred Heart Hospital, Anyang, Korea

Address for correspondence:

Dr. Young Kyung Lee

Department of Laboratory Medicine,

Hallym University Sacred Heart Hospital, Anyang, Korea

Tel: +82-31-380-3930

Fax: +82-31-380-3934

E-mail: lyoungk@hallym.or.kr

Supplementary Material

Supplementary Methods

Search Strategies on Medline, EMBASE and Cochrane Central

Characteristics of the Excluded Study

Supplementary Tables

Supplementary Table 1. Summary of pre-analytical conditions

Supplementary Table 2. Checklist of items to include when reporting a systematic review or meta-analysis (PRISMA guidelines)

Supplementary Table 3. The Newcastle-Ottawa Scale for assessing the quality of non-randomized studies in meta-analysis

Supplementary Figure

Supplementary Figure 1. Funnel plots for evaluation of publication bias

Supplementary Methods

Search Strategies

Pubmed*			EMBASE†			Cochrane Library		
						**#38**	#37 with "trials"	**86**
**#37**	#10 AND #36	**2293**	**#37**	#10 AND #36	**4497**	**#37**	#10 AND #36	**103**
**#36**	#28 OR #35	**24164**	**#36**	#28 OR #35	**44727**	**#36**	#28 OR #35	**4261**
**#35**	#29 OR #30 OR #31 OR #32 OR #33 OR #34	**113**	**#35**	#29 OR #30 OR #31 OR #32 OR #33 OR #34	**33723**	**#35**	#29 OR #30 OR #31 OR #32 OR #33 OR #34	**1074**
**#34**	kirsten rat sarcoma viral oncogene	**441**	**#34**	kirsten rat sarcoma viral oncogene	**475**	**#34**	kirsten rat sarcoma viral oncogene	**31**
**#33**	v-Ki-ras2	**157**	**#33**	v-Ki-ras2	**170**	**#33**	v-Ki-ras2	**10**
**#32**	v-Ki-ras	**147**	**#32**	v-Ki-ras	**150**	**#32**	v-Ki-ras	**0**
**#31**	Ki-ras	**1363**	**#31**	Ki-ras	**1423**	**#31**	Ki-ras	**5**
**#30**	K-ras	**6025**	**#30**	K-ras	**8713**	**#30**	K-ras	**471**
**#29**	KRAS	**10731**	**#29**	KRAS	**24327**	**#29**	KRAS	**1024**

**#28**	#11 OR #12 OR #13 OR #14 OR #15 OR #16 OR #17 OR #18 OR #19 OR #20 OR #21 OR #22 OR #23 OR #24 OR #25 OR #26 OR #27	**6626**
**#27**	cftDNA	**11**
**#26**	circulating free tumour DNA	**101**
**#25**	circulating free tumor DNA	**25**
**#24**	ctDNA	**1330**
**#23**	circulating tumour DNA	**163**
**#22**	circulating tumor DNA	**1128**
**#21**	cfDNA	**1253**
**#20**	cell-free circulating DNA	**103**
**#19**	circulating free DNA	**173**
**#18**	circulating cell-free DNA	**570**

**#28**	#11 OR #12 OR #13 OR #14 OR #15 OR #16 OR #17 OR #18 OR #19 OR #20 OR #21 OR #22 OR #23 OR #24 OR #25 OR #26 OR #27	**12123**
**#27**	cftDNA	**32**
**#26**	circulating free tumour DNA	**13**
**#25**	circulating free tumor DNA	**56**
**#24**	ctDNA	**2773**
**#23**	circulating tumour DNA	**348**
**#22**	circulating tumor DNA	**2351**
**#21**	cfDNA	**2920**
**#20**	cell-free circulating DNA	**177**
**#19**	circulating free DNA	**346**
**#18**	circulating cell-free DNA	**1094**

**#28**	#11 OR #12 OR #13 OR #14 OR #15 OR #16 OR #17 OR #18 OR #19 OR #20 OR #21 OR #22 OR #23 OR #24 OR #25 OR #26 OR #27	**3258**
**#27**	cftDNA	**1**
**#26**	circulating free tumour DNA	**166**
**#25**	circulating free tumor DNA	**166**
**#24**	ctDNA	**96**
**#23**	circulating tumour DNA	**297**
**#22**	circulating tumor DNA	**298**
**#21**	cfDNA	**71**
**#20**	cell-free circulating DNA	**62**
**#19**	circulating free DNA	**247**
**#18**	circulating cell-free DNA	**62**

**#17**	cell-free DNA	**2624**
**#16**	cell free DNA	**2624**
**#15**	circulating plasma DNA	**53**
**#14**	circulating DNA	**787**
**#13**	circulating nucleic acids	**229**
**#12**	plasma DNA	**895**
**#11**	liquid biopsy	**1735**
**#10**	#1 OR #2 OR #3 OR #4 OR #5 OR #6 OR #7 OR #8 OR #9	**45103**
**#9**	PDAC	**4284**
**#8**	pancreatic ductal adenocarcinomas	**638**
**#7**	pancreatic ductal adenocarcinoma	**6299**
**#6**	pancreatic adenocarcinomas	**946**

**#17**	cell-free DNA	**4740**
**#16**	cell free DNA	**4740**
**#15**	circulating plasma DNA	**78**
**#14**	circulating DNA	**1335**
**#13**	circulating nucleic acids	**340**
**#12**	plasma DNA	**1571**
**#11**	liquid biopsy	**3181**
**#10**	#1 OR #2 OR #3 OR #4 OR #5 OR #6 OR #7 OR #8 OR #9	**72070**
**#9**	PDAC	**8468**
**#8**	pancreatic ductal adenocarcinomas	**969**
**#7**	pancreatic ductal adenocarcinoma	**10836**
**#6**	pancreatic adenocarcinomas	**1297**

**#17**	cell-free DNA	**151**
**#16**	cell free DNA	**1071**
**#15**	circulating plasma DNA	**212**
**#14**	circulating DNA	**583**
**#13**	circulating nucleic acids	**18**
**#12**	plasma DNA	**1792**
**#11**	liquid biopsy	**395**
**#10**	#1 OR #2 OR #3 OR #4 OR #5 OR #6 OR #7 OR #8 OR #9	**3639**
**#9**	PDAC	**160**
**#8**	pancreatic ductal adenocarcinomas	**17**
**#7**	pancreatic ductal adenocarcinoma	**275**
**#6**	pancreatic adenocarcinomas	**83**

**#5**	pancreatic adenocarcinoma	**6509**	**#5**	pancreatic adenocarcinoma	**10974**	**#5**	pancreatic adenocarcinoma	**1148**
**#4**	pancreatic carcinomas	**1005**	**#4**	pancreatic carcinomas	**1277**	**#4**	pancreatic carcinomas	**134**
**#3**	pancreatic carcinoma	**6346**	**#3**	pancreatic carcinoma	**8768**	**#3**	pancreatic carcinoma	**818**
**#2**	pancreatic cancers	**2563**	**#2**	pancreatic cancers	**3959**	**#2**	pancreatic cancers	**381**
**#1**	pancreatic cancer	**33967**	**#1**	pancreatic cancer	**55256**	**#1**	pancreatic cancer	**3075**

[*] Search options were limited to title or abstract by using commands as shown: ([Title/Abstract])

[†] Search options were limited to title, keyword, or abstract by using commands as shown: ([Title/Keyword/Abstract] : “:ti,ab,kw”)

Characteristics of the Excluded Studies

No.	Title	First Author	Journal	Main Reason for Exclusion
1	Detection of K-ras Gene Mutations in Plasma DNA of Patients with Pancreatic Adenocarcinoma: Correlation with Clinicopathological Features	Yamada (1998)	Clin Cancer Res	The number of resectable pancreatic cancer patients was too small to extract meaningful hazard ratios.
2	K-ras Mutations in DNA Extracted From the Plasma of Patients With Pancreatic Carcinoma: Diagnostic Utility and Prognostic Significance	Castells (1999)	J Clin Oncol	The number of resectable pancreatic cancer patients was too small to extract meaningful hazard ratios.
3	Follow-Up Study of K-ras Mutations in the Plasma of Patients With Pancreatic Cancer Correlation With Clinical Features and Carbohydrate Antigen 19-9	Dabritz (2009)	Pancreas	The number of resectable pancreatic cancer patients was too small to extract meaningful hazard ratios.
4	K-ras mutational status predicts poor prognosis in unresectable pancreatic cancer	Chen (2010)	Eur J Surg Oncol	The study population included only unresectable (advanced) or metastatic pancreatic cancer patients.
5	Circulating tumor cells (CTC) and KRAS mutant circulating free DNA (cfDNA) detection in peripheral blood as biomarkers in	Earl (2015)	BMC Cancer	The number of resectable pancreatic cancer patients was too small to extract meaningful hazard ratios.

	patients diagnosed with exocrine pancreatic cancer			
6	Detection of K-ras Gene Mutation by Liquid Biopsy in Patients With Pancreatic Cancer	Kinugasa (2015)	Cancer	The number of resectable pancreatic cancer patients was too small to extract meaningful hazard ratios.
7	Clinical implications of genomic alterations in the tumour and circulation of pancreatic cancer patients	Sausen (2015)	Nat Commun	The study was not related with our topic.
8	High Levels of Cell-Free Circulating Nucleic Acids in Pancreatic Cancer are Associated With Vascular Encasement, Metastasis and Poor Survival	Singh (2015)	Cancer Invest	We requested additional data for the patients with resectable stage, but we did not receive any response from the authors.
9	Pharmacodynamic separation of gemcitabine and erlotinib in locally advanced or metastatic pancreatic cancer: therapeutic and biomarker results	Semrad (2015)	Int J Clin Oncol	The study population included only unresectable (advanced) or metastatic pancreatic cancer patients.
10	Clinical relevance of circulating KRAS mutated DNA in plasma from patients with advanced pancreatic cancer	Tjensvoll (2016)	Mol Oncol	The study population included only unresectable (advanced) or metastatic pancreatic cancer patients.
11	Ultrasensitive plasma ctDNA KRAS assay for detection, prognosis, and assessment of	Chen (2017)	Oncotarget	The study population included only unresectable (advanced) or metastatic

	therapeutic response in patients with unresectable pancreatic ductal adenocarcinoma			pancreatic cancer patients.
12	Analysis of ctDNA to predict prognosis and monitor treatment responses in metastatic pancreatic cancer patients	Cheng (2017)	Int J Cancer	The study population included only unresectable (advanced) or metastatic pancreatic cancer patients.
13	Profiling tumour heterogeneity through circulating tumour DNA in patients with pancreatic cancer	Adamo (2017)	Oncotarget	The number of resectable pancreatic cancer patients was too small to extract meaningful hazard ratios.
14	Early changes in plasma DNA levels of mutant KRAS as a sensitive marker of response to chemotherapy in pancreatic cancer	Del (2017)	Sci Rep	The study population included only unresectable (advanced) or metastatic pancreatic cancer patients.
15	A pilot study evaluating concordance between blood-based and patient-matched tumor molecular testing within pancreatic cancer patients participating in the Know Your Tumor (KYT) initiative	Pishvaian (2017)	Oncotarget	The hazard ratios and confidence intervals or p-values were not presented as an analyzable form.
16	Diagnostic value of CA19.9, circulating tumour DNA and circulating tumour cells in patients with solid pancreatic tumours	Sefrioui (2017)	Br J Cancer	The study was not related with our topic.

17	Phase I/II Study of Refametinib (BAY 86-9766) in Combination with Gemcitabine in Advanced Pancreatic cancer	Laethem (2017)	Targ Oncol	The study population included only unresectable (advanced) or metastatic pancreatic cancer patients.
18	Mutant KRAS Circulating Tumor DNA Is an Accurate Tool for Pancreatic Cancer Monitoring	Perets (2018)	Oncologist	The study population included only unresectable (advanced) or metastatic pancreatic cancer patients.
19	Circulating Tumor DNA as a Sensitive Marker in Patients Undergoing Irreversible Electroporation for Pancreatic Cancer	Lin (2018)	Cell Physiol Biochem	The number of resectable pancreatic cancer patients was too small to extract meaningful hazard ratios.
20	Circulating Nucleic Acids Are Associated With Outcomes of Patients With Pancreatic Cancer	Bernard (2018)	Gastroenterology	The study was not related with our topic.

Supplementary Tables

Supplementary Table 1. Summary of pre-analytical conditions

Author	Year	Blood collection tubes	Blood volume	Time to process after blood collection	Plasma or serum preparation	cfDNA isolation method
Takai et al.	2015	EDTA tubes	NA	Immediately	10 min at 1,600*g* (4 ℃)<br>+ 10 min at 16,000*g* (4 ℃)	QIAamp Circulating Nucleic Acid kit (Qiagen)
Hadano et al.	2016	EDTA tubes	8 mL	Within 4h	10 min at 1,500*g* (RT)	QIAamp Circulating Nucleic Acid kit (Qiagen)
Pietrasz et al.	2017	EDTA tubes	9 mL	Within 3h	15 min at 3,500rpm (4 ℃)	QIAamp Circulating Nucleic Acid kit (Qiagen)
Kim et al.	2018	NA	NA	Within 2h	10 min at 1,800*g* (4 ℃)<br>+ 10 min at 16,000*g* (4 ℃)	QIAamp Circulating Nucleic Acid kit (Qiagen)
Nakano et al.	2018	Venoject II® tubes	12 mL	Immediately	10 min at 1,750*g* (21 ℃)	Plasma/Serum Cell-Free Circulating DNA Purification Midi Kit (Norgen Biotek)

Abbreviations: NA, not available; RT, room temperature

Supplementary Table 2. The Newcastle-Ottawa Scale for assessing the quality of non-randomized studies in meta-analysis

No.	Author	Year	Selection	Comparability	Outcome
1	Takai et al.	2015	★★★★	★★	★★★
2	Hadano et al.	2016	★★★★	★★	★★★
3	Pietrasz et al.	2017	★★★★	★★	★★★
4	Kim et al.	2018	★★★★	★★	★★★
5	Nakano et al.	2018	★★★★	★	★★★

The Newcastle-Ottawa Scale

Selection (Maximum of one star for each numbered item)

1. Representativeness of the exposed cohort
   a) truly representative of the average ___________ (describe) in the community *
   b) somewhat representative of the average ___________ in the community *
   c) selected group of users eg nurses, volunteers
   d) no description of the derivation of the cohort
2. Selection of the non exposed cohort
   a) drawn from the same community as the exposed cohort *
   b) drawn from a different source
   c) no description of the derivation of the non exposed cohort
3. Ascertainment of exposure to implants
   a) secure record (eg surgical records) *
   b) structured interview *
   c) written self report
   d) no description
4. Demonstration that outcome of interest was not present at start of study
   a) yes * b) no

Comparability (Maximum of two stars for comparability)

1. Comparability of cohorts on the basis of the design or analysis

a) study controls for ___________ (select the most important factor) *
b) study controls for any additional factor (This criteria could be modified to indicate specific control for a second important factor.) *

Outcome (Maximum of one star for each numbered item)

1. Assessment of outcome
   a) independent blind assessment *
   b) record linkage *
   c) self report
   d) no description

2. Was follow up long enough for outcomes to occur
   a) yes * b) no

3. Adequacy of follow-up of cohorts
   a) complete follow up - all subjects accounted for *
   b) subjects lost to follow up unlikely to introduce bias *
   c) follow up rate < ___% and no description of those lost
   d) no statement

Supplementary Table 3. Checklist of items to include when reporting a systematic review or meta-analysis (PRISMA guidelines)

Section/topic		Checklist item	Reported on page #
**TITLE**			
Title	1	Identify the report as a systematic review, meta-analysis, or both.	1
**ABSTRACT**			
Structured summary	2	Provide a structured summary including, as applicable: background; objectives; data sources; study eligibility criteria, participants, and interventions; study appraisal and synthesis methods; results; limitations; conclusions and implications of key findings; systematic review registration number.	3
**INTRODUCTION**			
Rationale	3	Describe the rationale for the review in the context of what is already known.	5
Objectives	4	Provide an explicit statement of questions being addressed with reference to participants, interventions, comparisons, outcomes, and study design (PICOS).	5
**METHODS**			
Protocol and registration	5	Indicate if a review protocol exists, if and where it can be accessed (e.g., Web address), and, if available, provide registration information including registration number.	6
Eligibility criteria	6	Specify study characteristics (e.g., PICOS, length of follow-up) and report characteristics (e.g., years considered, language, publication status) used as criteria for eligibility, giving rationale.	6
Information sources	7	Describe all information sources (e.g., databases with dates of coverage, contact with study authors to identify	6

Section/topic		Checklist item	Reported on page #
		additional studies) in the search and date last searched.	
Search	8	Present full electronic search strategy for at least one database, including any limits used, such that it could be repeated.	6 and Supplementary Methods
Study selection	9	State the process for selecting studies (i.e., screening, eligibility, included in systematic review, and, if applicable, included in the meta-analysis).	6
Data collection process	10	Describe method of data extraction from reports (e.g., piloted forms, independently, in duplicate) and any processes for obtaining and confirming data from investigators.	6-7
Data items	11	List and define all variables for which data were sought (e.g., PICOS, funding sources) and any assumptions and simplifications made.	7
Risk of bias in individual studies	12	Describe methods used for assessing risk of bias of individual studies (including specification of whether this was done at the study or outcome level), and how this information is to be used in any data synthesis.	7 and Supplementary Methods
Summary measures	13	State the principal summary measures (e.g., risk ratio, difference in means).	7-8
Synthesis of results	14	Describe the methods of handling data and combining results of studies, if done, including measures of consistency (e.g., I^2) for each meta-analysis.	7
Risk of bias across studies	15	Specify any assessment of risk of bias that may affect the cumulative evidence (e.g., publication bias, selective reporting within studies).	7-9 and Supplementary Table 2
Additional analyses	16	Describe methods of additional analyses (e.g., sensitivity or subgroup analyses, meta-regression), if done, indicating which were pre-specified.	7-8

Section/topic		Checklist item	Reported on page #
**RESULTS**			
Study selection	17	Give numbers of studies screened, assessed for eligibility, and included in the review, with reasons for exclusions at each stage, ideally with a flow diagram.	8 and Figure 1
Study characteristics	18	For each study, present characteristics for which data were extracted (e.g., study size, PICOS, follow-up period) and provide the citations.	8-9 and Table 1
Risk of bias within studies	19	Present data on risk of bias of each study and, if available, any outcome-level assessment (see Item 12).	8-9 and Supplementary Table 2
Results of individual studies	20	For all outcomes considered (benefits or harms), present, for each study: (a) simple summary data for each intervention group and (b) effect estimates and confidence intervals, ideally with a forest plot.	9
Synthesis of results	21	Present results of each meta-analysis done, including confidence intervals and measures of consistency.	9, Figure 2-3
Risk of bias across studies	22	Present results of any assessment of risk of bias across studies (see Item 15).	7-8, Supplmentary Figure 1
Additional analysis	23	Give results of additional analyses, if done (e.g., sensitivity or subgroup analyses, meta-regression [see Item 16]).	10, Figure 4-5
**DISCUSSION**			
Summary of evidence	24	Summarize the main findings including the strength of evidence for each main outcome; consider their relevance to key groups (e.g., health care providers, users, and policy makers).	10
Limitations	25	Discuss limitations at study and outcome level (e.g., risk of bias), and at review level (e.g., incomplete retrieval of identified research, reporting bias).	13

Section/topic		Checklist item	Reported on page #
Conclusions	26	Provide a general interpretation of the results in the context of other evidence, and implications for future research.	13
**FUNDING**			
Funding	27	Describe sources of funding for the systematic review and other support (e.g., supply of data); role of funders for the systematic review.	14

Supplementary Figure

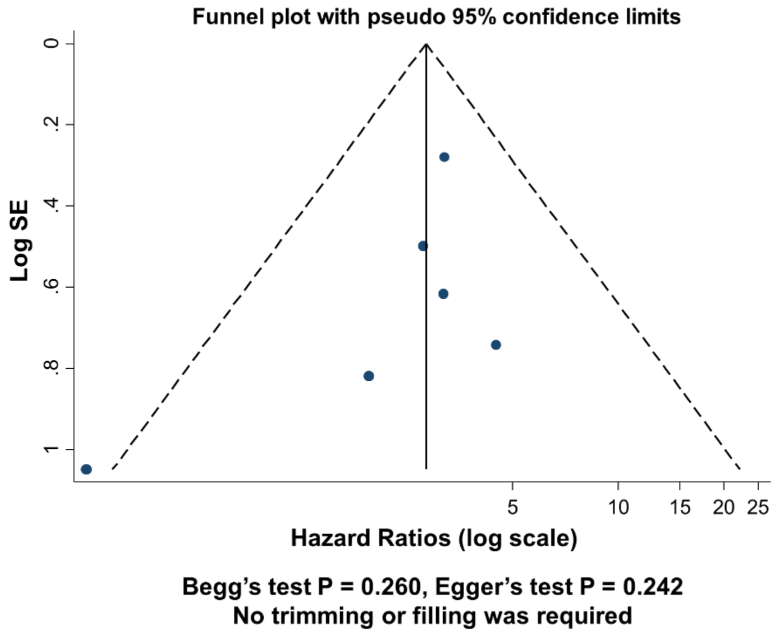

Supplementary Figure 1. Funnel plots for evaluation of publication bias
The results of Egger's and Begg's tests are presented. Using the trim-and-fill method, no trimming was done due to absence of asymmetry.
Abbreviations: SE, standard error.
